# Strain Variation Based on Spike Glycoprotein Gene of SARS-CoV-2 in Kuwait from 2020 to 2021

**DOI:** 10.3390/pathogens11090985

**Published:** 2022-08-29

**Authors:** Nada Madi, Mohammad Sadeq, Sahar Essa, Hussain A. Safar, Anfal Al-Adwani, Marwa Al-Khabbaz

**Affiliations:** 1 Virology Unit, Department of Microbiology, Faculty of Medicine, Kuwait University, Safat 13110, Kuwait; 2 Jaber Al-Ahmad Armed Forces Hospital, Sabhan 91710, Kuwait; 3 Research Core Facility and OMICS Research Unit, Faculty of Medicine, Kuwait University, Safat 13110, Kuwait

**Keywords:** SARS-CoV-2, spike glycoprotein, strain variation, Kuwait

## Abstract

Severe acute respiratory syndrome coronavirus-2 (SARS-CoV-2) is the causative agent of coronavirus disease 2019 (COVID-19), which was first identified in Wuhan, China, in December 2019. With the global transmission of the virus, many SARS-CoV-2 variants have emerged due to the alterations of the spike glycoprotein. Therefore, the S glycoprotein encoding gene has widely been used for the molecular analysis of SARS-Co-2 due to its features affecting antigenicity and immunogenicity. We analyzed the S gene sequences of 35 SARS-CoV-2 isolates in Kuwait from March 2020 to February 2021 using the Sanger method and MinION nanopore technology to confirm novel nucleotide alterations. Our results show that the Kuwaiti strains from clade 19A and B were the dominant variants early in the pandemic, while clade 20I (Alpha, V1) was the dominant variant from February 2021 onward. Besides the known mutations, 21 nucleotide deletions in the S glycoprotein in one Kuwaiti strain were detected, which might reveal a recombinant SARS-CoV-2 with the defective viral genome (DVG). This study emphasizes the importance of closely perceiving the emerging clades with these mutations during this continuous pandemic as some may influence the specificity of diagnostic tests, such as RT-PCR and even vaccine design directing these positions.

## 1. Introduction

The novel severe acute respiratory syndrome coronavirus-2 (SARS-CoV-2), the causative agent of coronavirus disease (COVID-19), has become a global threat since its first identification in Wuhan City, China, in December 2020 [1]. At the time of writing, the death toll has surpassed 6 million among 596,020,485 confirmed cases around the Globe (until 21 August 2022) [2].

According to the phylogenetic analysis, SARS-CoV-2 is a member of the genus Betacoronavirus, including the SARS coronavirus (SARS-CoV) [3,4]. Studies have shown that SARS-CoV-2 has a close relationship with bat SARS-like CoVs [5]; however, the intermediate host for the transmission to humans remains uncertain. Many progenies of SARS-CoV-2 have already been produced from the original Wuhan strain due to a high number of mutations in the genome, thus escaping the host immune responses [6,7,8,9,10]. SARS-CoV-2 is an enveloped, single-stranded positive-sense RNA with a genome of approximately 29.9 kb encoding 27 proteins from 14 ORFs; 15 non-structural, eight accessory, and four major structural proteins [4]. The four major structural proteins—spike (S) protein, nucleoprotein (N), membrane (M) protein, and envelope (E) protein—are required for the completion of successful infection events of viral entry, assembly, packaging, and release of new viral particles inside human cells [11,12,13].

There is evidence that the spike (S) protein, a 1273 amino acid long glycoprotein with multiple domains, plays a significant role in SARS-CoV-2 pathogenesis. Like other coronaviruses, the S protein of SARS-CoV-2 facilitates receptor recognition, cell attachment, and fusion during viral infection [14]. It binds to the angiotensin-converting enzyme 2 (ACE2) receptor [15]. The S protein consists of two functional subunits, S1 and S2, with a critical rule in viral entry. The S1 subunit recognizes and binds to the host receptor through the receptor-binding domain (RBD), while S2 is responsible for fusion with the host cell membrane [16,17]. Moreover, recent studies have shown that neutralizing antibodies are generated in response to the entry and fusion of surface-exposed S protein (mainly RBD), making it an important target for vaccine development [18,19]. Several variants of SARS-CoV-2 have emerged depending on amino acid mutations in S gene sequences, and molecular analysis based on the S gene can deliver visions into antigenicity, immunogenicity, or evolutionary trends [20]. Therefore, in this study, we analyzed the S gene sequences of SARS-CoV-2 to understand this virus better. Furthermore, detecting and understanding the sequence variations of S glycoprotein among the circulating viruses is essential for better understanding the biology of SARS-CoV-2 infection, developing antiviral treatments, and designing potent vaccines.

In this study, we analyzed spike glycoprotein gene sequences of SARS-CoV-2 circulating in Kuwait to identify mutations causing amino acid variations in the glycoprotein S. The identification of the diversification in SARS-CoV-2 during the evolution of the virus is essential for the early diagnosis and control of the disease.

## 2. Results

### 2.1. Characteristics of the Patients

Between March 2020 and February 2021, 35 samples from SARS-CoV-2 positive patients were collected and analyzed. These samples consisted of residual nasopharyngeal swabs that tested positive for SARS-CoV-2 by RT-PCR. The average age of the enrolled patients was 46 years (range (2–91)), and the gender distribution was 49% (17/35) female and 51% (18/35) male, 54% (19/35) were Kuwaiti among the patients, and 46% (16/35) were non-Kuwaiti. Symptomatic individuals represented 60%, more than half of the study population. According to SARS-CoV-2 [21] guidelines, symptomatic patients enrolled in this study had mild to critical SARS-CoV-2 infection; yet, 14 (40%) of the patients were admitted to ICU, while two (5.7%) died. Details on the clinical conditions and symptoms of the symptomatic patients are presented in Table 1.

### 2.2. Identification of Mutations and Deletions in the SARS-CoV-2 Spike Glycoprotein Gene

Based on mutation analysis of the SARS-CoV-2 isolates, 32 of 35 isolates from Kuwait contained a signature 23063A>G (D614G) mutation in the spike glycoprotein, which represents the most frequent mutation (91%) (Table 2). The second frequent mutations found at a similar rate were 23271 C>A (A570D), 23604 C>A (P681H), and 24914 G>C (D1118H), found in 40% (14/35) of the isolates. On the other hand, deletions at nt position 21766–21771 (ACATGT); (H69-V70del) were the most frequent deletions, found in 40% (14/35) of the isolates in Kuwait, while the second most frequent deletion, which was found in 37% (13/35) of the isolates, was at nt position 21992–21994 (TAT); (Y144-del). Mutation at nt position 23709 C>T (T716I) and mutation at nt position 24506 T>G (S982A) were found less frequently (37%, 13/35) in the Kuwaiti isolates. In addition, single nucleotide–polymorphism (SNP) at position 23063 A>T (N501Y) was found in 34% (12/35) of the isolates, while SNP at position 22622 A>G (N354D) was the least frequent mutation detected in the isolates, found in only 6% (2/35) of the isolates.

Interestingly, two novel synonymous mutations (22498 C>T [G311G], 22051 G>T [D1163D]) were each found in only one isolate (3%). Additionally, novel and unique deletions at nt position 22331–22351delGGTTGGACAGCTGGTGCTGCA (G257-A263del) represented a defective SARS-CoV-2 stain and were detected in only one Kuwaiti isolate (Table 2). This unique deletion was confirmed with whole-genome sequencing of SARS-CoV-2 using MinION Nanopore technology. Raw data of the sequences, including the whole genome, are provided as Supplementary Materials.

### 2.3. Genomic Variations of SARS-CoV-2 Based on the S Glycoprotein Gene

**Figure 1:**
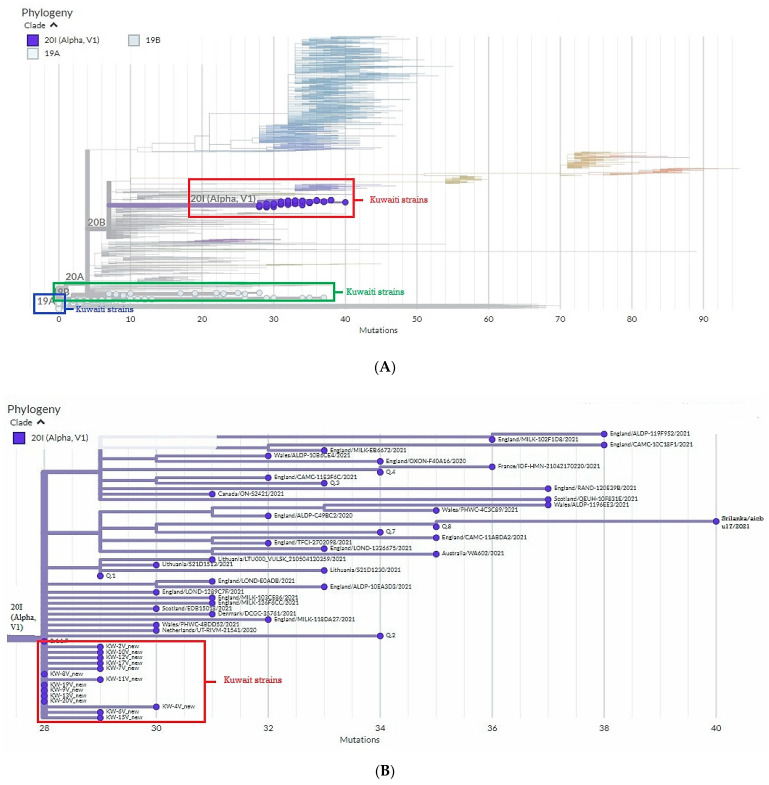

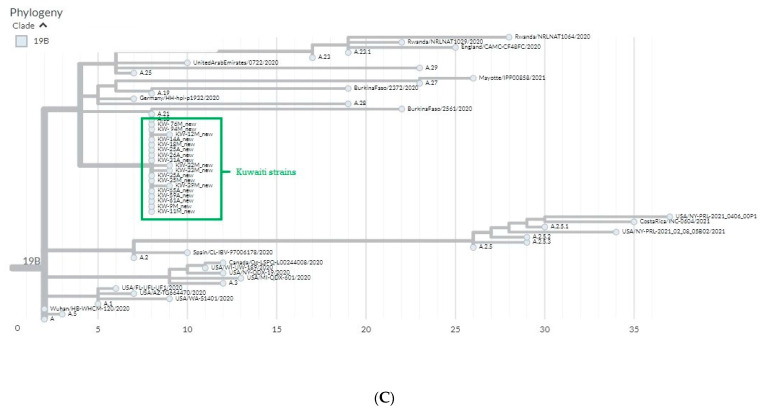
Phylogenetic tree of Kuwaiti sequences based on s gene sequencing compared to the global strains. (**A**) Maximum clade credibility tree constructed by Nextclade (https://clades.nextstrain.org/ accessed on 21 August 2022) of S gene sequences from Kuwait and all sequences available in GISAID. Global clades are colored differently, and clades corresponding to Kuwaiti strains are circled in red. (**B**) Divergence of Kuwait strains clade 20I (Alpha, V1) from 19A clade. (**C**) Divergence of Kuwait strains clade 19B from 19A clade.

To define the circulating clades of SARS-CoV-2 in Kuwait, we applied the Nextstrain nomenclature that includes the original clade 19A and its derivation 19B and the emerging clade 20B and its derivation 20I. The phylogenetic analytical tree of 35 SARS-CoV-2 isolates from Kuwait and the global strains based on the S glycoprotein gene was performed by Nextclade ^beta^ of Nextstrain platform, which is based on banded Smith–Waterman alignment with an affine gap-penalty. The evolutional distances showed that all the SARS-CoV-2 isolates circulating in Kuwait between March 2020 and March 2021 belong to three main clades: 19A, 19B, and 20I (Alpha/V1) (Figure 1A, Table 1). Clade 19B constituted 51% (18/35) of the Kuwaiti strains, while 40% (14/35) of the strains belonged to clade 20I (Alpha, V1), which was first identified in the UK in the fall of 2020. The least abundant clade was 19A (9%; 3/35), the original clade of SARS-CoV-2 identified in Wuhan in December 2019. As for the time evolution, the data showed that clades 19A and 19B were dominant from March to December 2020, while from February 2021 onwards, clade 20I was the dominant (Table 1). Figure 1B shows that there is a divergence of 28–30% at the nucleotides level from the original clade 19A (NC_045512) and the Kuwaiti strains of clade 20I/Alpha/501Y.V1, as well as 7–9% divergence in the nucleotides levels from clade 19A (NC_045512) and the Kuwaiti strains of clade 19B (Figure 1C).

**Figure 2:**
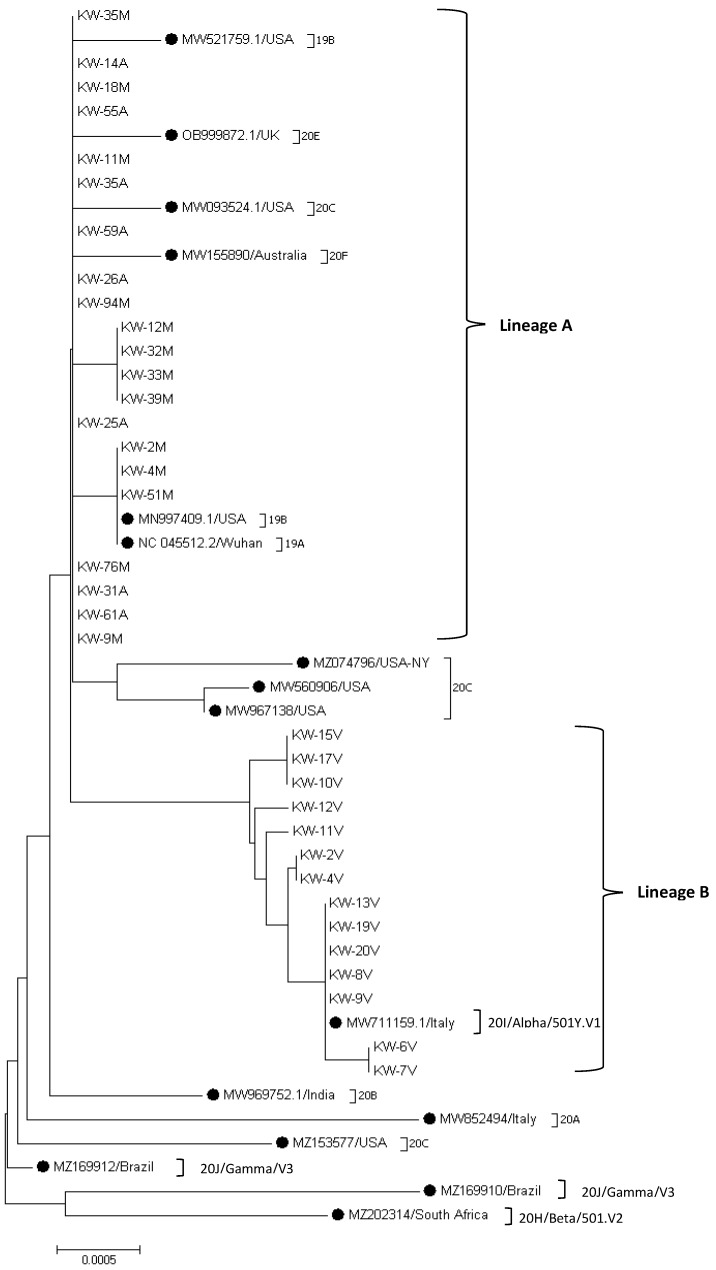
Sequence phylogeny of SARS-CoV-2 spike gene strains in Kuwait. Phylogenic tree representing SARS-CoV-2 circulated in Kuwait and other SARS-CoV-2 variants, retrieved from the NCBI database using the Neighbor-Joining method of MEGA 7 based on the Kimura 2-parameter method substitution model, and tree structure was confirmed by running analysis on 1000 bootstraps. The branch length is indicated in the scale bar. Reference strains are indicated by ●.

More detailed phylogenetic analysis of the Kuwaiti strains of SARS-CoV-2 based on spike glycoprotein encoded gene sequence grounded on the evolutional tree constructed by MEGA 7 software demonstrated that the Kuwaiti strains are clustered into two main lineages with nucleotide identity 98.8% between all Kuwaiti SARS-CoV-2 strains (Figure 2). Lineage A contained 21/35 (60%) of the strains that are subdivided into three sub-lineage; the first contained 14 Kuwaiti strains (KW-35M, KW-14A, KW-18M, KW-55A, KW-11M, KW-35A, KW-59A, KW-26A, KW-94M, KW-25A, KW-76M, KW-31A, KW-61A, KW-9M) that clustered with the reference strains; MW521759.1/USA (clade 19B), OB999872.1/UK (clade 20E), MW093524.1/USA (clade 20C), and MW155890/Australia (clade 20F) with 0–0.1% divergence among the strains, whereas the second sub-lineage contained four identical Kuwaiti strains (KW-12M, KW-32M, KW-33M, and KW-39 M) with 0–0.1% divergence at the nucleotide level with MN997409.1/USA (clade 19B) and NC_045512.2/Wuhan (clade 19A). The third sub-lineage of lineage A compromised three Kuwaiti strains (KW-2M, KW-4M, KW-51M) that were 100% identical at the nucleotide level to the reference strains MN997409.1/USA (clade 19B) and NC_045512.2/Wuhan (clade 19A) (Figure 2). Lineage B contained 14 Kuwaiti strains subdivided into four sub-lineages that showed 0–0.2% divergence at the nucleotide level with clade 20I/Alpha/501Y.V1.

### 2.4. Detection of 21 nt Deletion in S Glycoprotein Gene of SARS-CoV-2

**Figure 3:**
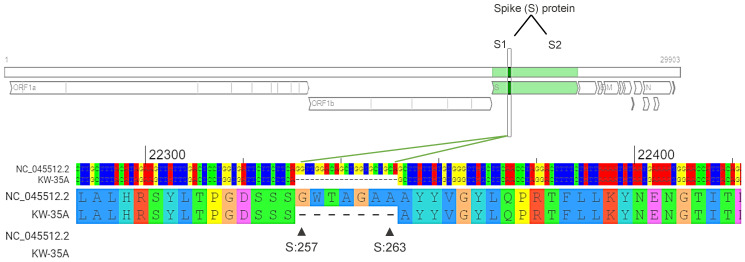
Deletions identified in the SARS-CoV-2 strain (KW-35A). High-throughput sequencing of the KW-35A strain revealed 21 nt deletions (22331-22351/GGTTGGACAGCTGGTGCTGCA) in the S gene corresponding to aa deletions (G257-A263) in spike glycoprotein. NC-045512.2 was used as a reference strain. Diagram was generated by the Genome Detective Coronavirus Typing Tool Version 1.13 (https://www.genomedetective.com/app/typingtool/cov/ accessed on 21 August 2022).

One Kuwaiti strain (KW-35A) showed a substantial deletion of 21 nucleotides at positions 22,331–22,351 (GGTTGGACAGCTGGTGCTGCA), which resulted in the deletion of amino acids G257–A263. This deletion is in the S1 subunit/N-terminal domain (14–305) of the spike glycoprotein (Figure 3). To eliminate the possibility that these findings were caused by errors in PCR amplification or Sanger sequencing, whole-genome sequencing of this strain was performed using the Oxford Nanopore Technologies MinION platform. The sequencing results confirmed these deletions, which may suggest the presence of a recombinant SARS-CoV-2 strain with a defective viral genome (DVG).

## 3. Discussion

This study, to our knowledge, is the first in Kuwait on the SARS-CoV-2 strain variation circulating in Kuwait from March 2020 to February 2021 based on the analysis of spike protein sequences. The first cases of SARS-CoV-2 were documented in February 2020 from 10 travelers arriving from Iran. After that, more cases were imported from other parts of the world, mainly from Europe (Italy), followed by additional importations from other European countries and worldwide until March 2020, when most air traffic was suspended. The analysis of the study population demographics indicates that males averaging 46 years showed higher positivity for SARS-CoV-2. Generally, SARS-CoV-2 infection causes flu-like symptoms; however, the symptoms may become severe and lead to ICU admission, whereas others die from complications [22]. Our study showed that fever, cough, pneumonia, SOB, reduced oxygen saturation, and bilateral infiltration of the lungs through X-ray imaging were frequent events among the admitted symptomatic patients. Patients admitted to the ICU had more severe symptoms that required intubation, and some died from complications of COVID-19 (Table 1). Those tested positive patients at the drive-through and lodged in the quarantine stations were asymptomatic and not followed up; therefore, it was unknown if they developed symptoms afterwards.

To explore SARS-CoV-2 variations, we identified positions in the spike glycoprotein-coding gene that were frequently altered and were known clade-defining positions in addition to novel positions detected for the first time in the Kuwaiti strains. According to the sequence analysis of the spike glycoprotein-encoding gene, the most frequent missense mutation (91%) identified in Kuwaiti strains was at nucleotide position 23,403, resulting in A>G alteration and causing an amino acid change from aspartate to glycine residue at position 614 (D614G). This mutation was frequently found globally (frequency in GISAID; 97.7%) and was either a solo mutation or combined with other mutations in 31 Kuwaiti strains from clades 19B and 20I (Alpha/V1). D614G is located in B-cell epitopes of the S1 subunit that contain numerous major and minor neutralizing antibody epitopes [16,23]. These amino acid changes are believed to alter immunogenicity, which results in the elimination of the B-cell epitopes. B-cells, however, play a significant role in recognizing pathogens and stimulating adaptive immunity in the immune response against virus infection. Therefore, this elimination will likely reduce the immunogenicity by obstructing the immune cell recognition of the virus [24]. Korber et al. showed that this mutation is associated with greater infectivity and higher viral loads but does not appear to cause severe disease [25]. Our results have also shown other missense mutations and deletions that were detected at relatively similar rates and were found as the second most frequent alterations; 23271 C>A (A570D), 23604 C>A (P681H), 24914 G>C (D1118H), 23709 C>T (T716I), 24506 T>G (S982A), 23063 A>T (N501Y), (H69-V70del), and (Y144-del) (Table 2). The least frequent mutations detected were: the missense mutation 22622 A>G (N354D), two novel synonymous mutations 22498 C>T (G311G) and 22051 G>T (D1163D), and the novel 21 nucleotide deletions (G257-A263).

It is well known that any mutation in the S gene coding for one of the structural proteins will affect the attachment of the virus and the transmission of the disease [26]. The previous alterations were distributed along with the spike protein, including the N-terminal domain (NTD) (14–305 residues), receptor-binding domain (RBD, 319–541 residues), heptapeptide repeat sequence 1 (HR1) (912–984 residues), and HR2 (11163–1213 residues) [27,28]. The S protein binds to ACE-2 (angiotensin-converting enzyme 2) via the NTD and RBD region of the S1 subunit, facilitating viral attachment to host cells in the form of a trimer; therefore, alterations in this position might alter the interaction of viral RBD with the host receptor [4,29]. HR1, on the other hand, is found in the S2 domain and is responsible for viral fusion and entry by forming helical bundles with HR2. Mutations in the HR1 region are predominantly responsible for conferring resistance to mouse hepatitis coronaviruses against HR2-derived peptide entry inhibitors [4,30].

Sequencing and analysis of the SARS-CoV-2 spike glycoprotein gene of SARS-CoV-2 strains revealed that different clades were circulated in Kuwait between March 2020 and February 2021, including 19A, 19B, and 20I (Alpha, V1). Of note, clades 19A and 19B emerged in Wuhan and have dominated worldwide, including Kuwait in the early outbreak, while 20I emerged over the summer of 2020 and included two “variants of concern” (VOC) with signature mutations S: N501Y. However, clade 20I quickly became dominant in Kuwait in February 2021, similar to observations across Europe and other parts of the world (Figure 1A, Table 1). Clade 19B, which was dominant in Kuwait from March to December 2020, characterized by the spike D614G amino acid changes, was the only site identified in early March 2020. At that time, the D614G mutation was rare globally and in Kuwait but gained distinction and became dominant afterwards. Before March 1, 2020, it was found in 10% of 997 global sequences; between March 1 and March 31, 2020, it represented 67% of 14,951 sequences, and between April 1 and May 18, 2020, it represented 78% of 12,194 sequences [25], however, until June 2021, it represented 99.7% of the global sequences (according to GISAID data).

According to Nextclade sequence analysis, the 19B variant in Kuwait showed 7–9% divergence at the nucleotide level from the original clade 19A (Figure 1C). Clade 20I (Alpha, V1) was detected as a new variant from clade 20B in Wales, the United Kingdom (UK), in September 2020, and it was detected for the first time in Kuwait in January 2021 in 11 travelers from the UK, which then became the dominant clade in Kuwait until May 2021 when the new variant 20A (Delta) started to emerge (data are not shown). Of note, clade 20I (Alpha, V1) is defined by many genetic changes, with at least 24 mutations, including 14 non-synonymous mutations, four deletions, and six synonymous mutations in ORF1ab, ORF8, nucleocapsid, and spike proteins [31]. In addition, sequence analysis based on the Nextclade platform demonstrated that 20I (Alpha, V1) variant in Kuwait showed 28–30% divergence at the nucleotide level from the original clade 19A (Figure 1B). Much evidence indicates that clade 20I is more efficiently transmitted than other SARS-CoV-2 variants circulating in the UK, and index patients with 20I infections are infected with a higher proportion of contacts than index patients infected with other variants [32,33]. Currently, there are no known differences in clinical outcomes associated with the SARS-CoV-2 variants; however, a higher transmission rate will lead to more detected cases, increasing the number of persons who need clinical care schemes, resulting in more deaths.

Genetic variability among Kuwaiti SARS-CoV-2 strains was also studied. The analysis demonstrated that 16 out of 3822 nucleotide positions were variable, with five (31%) being singleton sites and 11 (69%) positions being parsimony-informative sites. Our data have also shown 98.8% nucleotide identity between all Kuwait strains. The Kuwaiti strains were divided into two main lineages; the first lineage showed 0–0.1% divergence at the nucleotide level from clade 19A and 19B, while the second lineage showed 0–0.2% divergence at the nucleotide level with clade 20I (Alpha).

Remarkably, substantial deletions in one SARS-CoV-2 strain were detected using whole-genome sequencing with the Oxford Nanopore Technologies MinION platform. The strain contained multiple deletions at position 22,331–22,351 in the spike glycoprotein gene while retaining intact 5′ and 3′ genomic untranslated regions (5′ and 3′ UTRs). These deletions may suggest the presence of a recombinant strain with DVG. However, to support this assumption, in vitro assays must be performed. DVGs of SARS-CoV-2 are believed to be amplified by coronavirus replication-transcription complex (RTC) machinery supplied by co-infecting full-length helper coronaviruses [34,35]. Additionally, DVGs in respiratory viruses can act as pathogen-associated molecular patterns (PAMPs) and stimulate the innate immune system [36]. So far, the biological role of DVGs is mainly unknown, though some DVGs interfere with viral replication [37,38]. Therefore, coronaviruses infrequently perform recombination during their replication, producing complex populations of recombined RNA molecules.

## 4. Materials and Methods

### 4.1. Study Population

Thirty-five nasopharyngeal swab samples from confirmed cases of SARS-CoV-2 were collected between March 2020 and February 2021 by two hospitals in Kuwait, Al-Adan and Mubarak Al-Kabeer hospitals. Diagnostic purposes for the presence of SARS-CoV-2, based on real-time reverse transcriptase PCR assays (RT-PCR), were followed as per the Ministry of Health guidelines in Kuwait. Swab samples from Al-Adan hospital were collected from patients admitted to the ICU for COVID-19 complications. Swab samples collected from Mubarak Al-Kabeer hospital were from three sources; patients admitted to the hospital’s wards and ICU, individuals screened for SARS-CoV-2 infection in the Sabhan drive-through station for COVID-19 testing, and individuals screened for SARS-CoV-2 infection lodged in quarantine stations. The swab samples were sent to Mubarak Al-Kabeer hospital for SARS-CoV-2 RT-PCR testing.

### 4.2. S Gene Sequencing and Sequence Analysis

The complete length spike glycoprotein gene from the confirmed cases of SARS-CoV-2 was sequenced and analyzed. For this analysis, the original specimens from the patients were sequenced using the Sanger method. The PCR amplification and DNA sequencing of the entire length of the S gene was achieved using eight pairs of in-house designed primers (Table S1) [39]. The amplification profile comprised an initial denaturation at 95 °C for 5 min, followed by 35 amplification cycles (94 °C for 1 min, 65 °C for 1 min, and 72 °C for 2 min), with a final extension at 72 °C for 10 min. The amplicons were analyzed by electrophoresis in a 1.5% (W/V) agarose gel. The PCR products were purified with a QIAquick gel extraction kit (Qiagen, Hilden, Germany) according to the manufacturer’s instructions. Subsequently, the purified products were sequenced in the forward and the reverse directions by the primers. Sequencing was performed using the ABI 3500/3500× L genetic analyzer (PE Applied Biosystems, Inc., Foster City, CA, USA) with the ABI Prism BigDye Terminator Cycle Sequencing Ready Reaction kit (PE Applied Biosystems, Inc., Foster City, CA, USA). Mutations specific to the Kuwaitis SARS-CoV-2 isolates were identified using multiple analysis tools using the Wuhan SARS-CoV-2 virus as a reference sequence (NC_045512). Sequence variations analysis of Kuwaiti isolates’ S glycoprotein gene sequences and the reference genome was performed using the Clustal W method of MEGA software version 7. Clade assignment, mutation calling, and sequence quality checks were performed using the Nextstrain platform (https://nextstrain.org/, accessed on 21 August 2022) and CoVsurver mutations App-enabled by GISAID (https://www.gisaid.org/, accessed on 21 August 2022). Genome Detective Coronavirus Typing Tool Version 1.13 (https://www.genomedetective.com/app/typingtool/cov/, accessed on 21 August 2022) [40] was also used for variant analysis of SARS-CoV-2 isolates based on S gene sequences. The phylogenic tree was constructed using the Neighbor-Joining method of MEGA 7 based on the Kimura 2-parameter method as a substitution model with uniform rates among sites and complete gap deletion with 1000 replicates. Bootstrap pre-sampling (1000 replications) was used to measure the reliability of individual nodes in the phylogenetic tree. As for comparative phylogenetic analysis, different SARS-CoV-2 variants sequences published in the National Centre for Biotechnology Information (NCBI) database were used. Spike protein gene sequences of the Kuwaiti SARS-CoV-2 are publicly available in GISAID with accession IDs in Table 1.

### 4.3. SARS-CoV-2 Whole-Genome Sequencing Using MinION Nanopore Technology

The whole genome of SARS-CoV-2 from the sample “KW-35A” was sequenced using the Oxford Nanopore sequencing technology following the ARTIC network protocol [41]. Briefly, viral RNA was reverse-transcripted with LunaScript™ RT SuperMix Kit (New England Biolabs, Ipswich, MA, USA), and the transcribed cDNA was used as the primary input for overlapping tiled PCR reactions using ARTIC Network V3 primer pools with Q5 Hot Start High-Fidelity DNA polymerase (New England Biolabs, Ipswich, MA, USA). The amplicons were cleaned up with AMPure XP beads (Beckman Coulter Diagnostics, CA, USA), and the library was prepared using the ligation sequencing kit (SQK-LSK109) from Oxford Nanopore Technologies (Oxford, UK). Then, the library was quantified using QUBIT 1X dsDNA HS Assay Kit (Invitrogen, Waltham, Massachusetts, United States), and 15 ng of the prepared library was loaded into Oxford Nanopore MinION SpotON Flow Cells FLO-MIN106D, R9.4.1 (Oxford Nanopore Technologies, Oxford, UK). The FastQ files generated by the Mk1C device were used for analysis following the ARTIC Network analysis workflow and EP2ME-lab. The generated consensus file was used to align the sequence of KW-35A with the reference strain (NC_045512) using MEGA software version 7, while sequence variations analysis and mutation detection were performed using the Nextstrain platform and CoVsurver mutations App-enabled by GISAID.

## 5. Conclusions

The SARS-CoV-2 pandemic has caused a considerable impact on the health and economy worldwide. Thus, understanding the genetic diversity of the virus has become a priority in the fight against the disease. Frequently mutated positions were identified in Kuwaiti strains, most correlated with known clade-defining mutations and observed in sequences worldwide. Most of these mutations were alterations that caused a change in the AA attribute group, which has a bigger chance of affecting the protein. Therefore, it is crucial to closely perceive the emerging clades with these mutations during this continuous pandemic as some may influence the specificity of diagnostic tests such as RT-PCR and even vaccine design directing these positions.

## Figures and Tables

**Table 1:** Details on the Kuwaiti SARS-CoV-2 strains identified in the study population.

Patient Identifier	Strain Number	Collection Date	Nationality	Age	Sex	Clinical Data	Location	GISAID. Accession No.	Virus Clade
1	KW-61A	8 March 2020	NK	55	M	Fever, cough, pneumonia, CXR: bilateral infiltration.	ICU-AH ^▲^	EPI_ISL_3276797	19B
2	KW-76M	23 March 2020	NK	57	M	Fever, cough, loss of taste and smell sensations, SOB, SPO2 80% in right atrium, CXR: B/L infiltrates.	ICU-MKH ^●^	EPI_ISL_3276865	19B
3	KW-31A	7 April 2020	NK	72	M	Fever, cough, pneumonia, CXR: bilateral infiltration.	ICU-AH	EPI_ISL_3298298	19B
4	KW-2M	9 May 2020	K	77	M	Severe productive cough, SOB, wheeze, SPO2 90% in right atrium, generalized fatigue and tiredness, sore throat, CXR: B/L infiltrates.	ICU-MKH	EPI_ISL_3298299	19A
5	KW-4M	10 May 2020	K	62	F	DM type 2, progressive SOB, SPO2 88% in right atrium, high BP, CXR: B/L lung infiltrates and haziness >50% more on the right side.	ICU-MKH	EPI_ISL_3298301	19A
6	KW-94M	18 May 2020	K	91	F	Reduced level of consciousness, generalized fatigue, vomiting, intubated. Ketoacidosis. CXR: fair entry B/L. Patient deceased.	ICU-MKH	EPI_ISL_3298302	19B
7	KW-9M	18 May 2020	NK	62	F	COPD, smoker, dry cough, fever, SPO2 72% on arrival, CXR: B/L infiltrates and pulmonary congestion.	ICU-MKH	EPI_ISL_3298311	19B
8	KW-18M	26 May 2020	NK	69	M	Para-umbilical hernia, fever, cough, SOB, SPO2 85% in right atraium, blood acidosis. CXR: diffuse B/L infiltrates more on the left side. Put on mechanical ventilation—patient deceased.	ICU-MKH	EPI_ISL_3298374	19B
9	KW-26A	23 June 2020	NK.	49	M	Fever, pneumonia, CXR: bilateral infiltration/Retroperotonial hematoma.	ICU-AH	EPI_ISL_3298375	19B
10	KW-25A	24 June 2020	NK.	59	M	Fever, cough, pneumonia, CXR: bilateral infiltration.	ICU-AH	EPI_ISL_3298376	19B
11	KW-11M	1 July 2020	K	43	F	Fever, SOB, noisy chest pain, cough (progressive, green sputum), SPO2 >95% right atrium, chest: B/L wheeze. CXR: B/L haziness.	Ward 28-MKH ^♣^	EPI_ISL_3298377	19B
12	KW-55A	27 July 2020	NK	55	F	Fever, cough, pneumonia, CXR: bilateral infiltration.	ICU-AH	EPI_ISL_3298379	19B
13	KW-12M	28 July 2020	K	34	F	Exacerbation of B.A., headache, fever, low BP. Patient on bronchodilators, Chest: Normal. CXR: Normal.	Ward 28-MKH	EPI_ISL_3298381	19B
14	KW-59A	31 July 2020	K	70	F	Fever, pneumonia, CXR: bilateral infiltration.	ICU-AH	EPI_ISL_3298686	19B
15	KW-33M	8 August 2020	K	84	M	COPD, HTN, ex-smoker. 9 days H/O SOB, dry cough, 2 days fever, SPO2 66% on arrival —> 98%, CXR: B/L infiltrates and pulmonary congestion.	Ward 28-MKH	EPI_ISL_3298688	19B
16	KW-32M	11 August 2020	NK	42	M	DM, HTN on treatment, headache, blurred vision, abdominal pain. Chest: Normal. CXR: Normal.	Ward 28-MKH	EPI_ISL_3298691	19B
17	KW-39M	11 August 2020	NK	37	F	Fever, 3 days cough, 1 day SOB, pleuritic chest pain, SPO2 87% in right atrium, CXR: B/L infiltrates.	Ward 28-MKH	EPI_ISL_3298763	19B
18	KW-35M	19 August 2020	K	36	F	Dry cough, SOB, chills, runny nose, no fever. CXR: B/L scattered infiltrates. Chest exam: B/L scattered expiratory wheeze.	Ward 28-MKH	EPI_ISL_3298765	19B
19	KW-51M	5 October 2020	K	62	F	Uncontrolled DM type 2, HTN, fever, cough productive of sputum, pleuritic chest pain, SOB, SPO 88% in right atrium, loss of taste. Chest: reduced AE B/L. CXR: B/L haziness all over. CT of chest: lung fibrotic-like findings.	ICU-MKH	EPI_ISL_3298767	19A
20	KW-35A	7 October 2020	NK	31	M	Cough, pneumonia, CXR: bill reticular/miliary opacities.	ICU-AH	EPI_ISL_3298769	19B
21	KW-14A	7 December 2020	NK	65	M	Fever, cough, pneumonia, SOB, desaturating.	ICU-AH	EPI_ISL_3301950	19B
22	KW-2V	2 February 2021	K	24	F	None	Sabhan-Drive through ^■^	EPI_ISL_3302245	20I (Alpha, V1)
23	KW-4V	3 February 2021	K	25	F	None	Quarantine lodge	EPI_ISL_3302485	20I (Alpha, V1)
24	KW-6V	3 February 2021	K	26	F	None	Quarantine lodge	EPI_ISL_3302700	20I (Alpha, V1)
25	KW-7V	4 February 2021	NK.	58	M	None	Quarantine lodge	EPI_ISL_3302935	20I (Alpha, V1)
26	KW-8V	4 February 2021	K	35	F	None	Sabhan-Drive through	EPI_ISL_3303157	20I (Alpha, V1)
27	KW-9V	4 February 2021	K	34	M	None	Sabhan-Drive through	EPI_ISL_3303380	20I (Alpha, V1)
28	KW-11V	4 February 2021	NK	38	F	None	Sabhan-Drive through	EPI_ISL_3303631	20I (Alpha, V1)
29	KW-12V	4 February 2021	K	8	M	None	Sabhan-Drive through	EPI_ISL_3303726	20I (Alpha, V1)
30	KW-13V	4 February 2021	K	33	F	None	Sabhan-Drive through	EPI_ISL_3303727	20I (Alpha, V1)
31	KW-15V	4 February 2021	K	16	M	None	Sabhan-Drive through	EPI_ISL_3303729	20I (Alpha, V1)
32	KW-10V	4 February 2021	K	2	M	None	Sabhan-Drive through	EPI_ISL_3303932	20I (Alpha, V1)
33	KW-17V	5 February 2021	NK	30	M	None	Quarantine lodge	EPI_ISL_3303933	20I (Alpha, V1)
34	KW-19V	5 February 2021	K	38	M	None	Sabhan-Drive through	EPI_ISL_3303934	20I (Alpha, V1)
35	KW-20V	5 February 2021	NK	30	F	None	Sabhan-Drive through	EPI_ISL_3303935	20I (Alpha, V1)

^▲^ Intensive Care Unit-Al-Adan hospital; ^●^ Intensive Care Unit-Mubarak Al-Kabeer hospital; ^♣^ Ward 28-Mubarak Al-Kabeer Hospital; ^■^ Sabhan Drive-through station for COVID-19 testing; NK: Non-Kuwaiti; K: Kuwaiti; SOB: Shortness of breath; DM: Diabetes Mellitus; SPO2: Oxygen saturation; BP: blood pressure; CXR: Chest x-ray; BA: Bronchial Asthma; HTN: Hypertension; COPD: Chronic obstructive pulmonary disease.

**Table 2:** Variants frequencies of 35 Kuwaiti SARS-CoV-2 isolates.

Genome Change	Amino Acid Change	Type of Mutation	Number of Isolates	Frequency in GISAID (%)
23403 A>G	D614G	Missense	32	97.7
23271 C>A	A570D	Missense	14	45
23604 C>A	P681H	Missense	14	47.1
24914 G>C	D1118H	Missense	14	44.1
21766-21771delACATGT	H69-V70del	In frame deletion	14	45.5, 44.8
23709 C>T	T716I	Missense	13	44.8
24506 T>G	S982A	Missense	13	44
21992-21994delTAT	Y144-del	In frame deletion	13	44.1
23063 A>T	N501Y	Missense	12	47.5
22622 A>G	N354D	Missense	2	0.01
22498 C>T	G311G	Synonymous	1	0
22051 G>T	D1163D	Synonymous	1	0
22331-22351delGGTTGGACAGCTGGTGCTGCA	G257-A263del	In frame deletion	1	0

## Data Availability

Upon request to the corresponding author.

## Supplementary Materials

The following supporting information can be downloaded at: https://www.mdpi.com/article/10.3390/pathogens11090985/s1, File S1: S glycoprotein gene sequences of the 35 SARS-CoV-2 Kuwaiti isolates; File S2: Whole genome sequence of KW-35A SARS-CoV-2 isolate; Table S1: S gene primers.
